# Ionizing Radiation Technologies for Vaccine Development - A Mini Review

**DOI:** 10.3389/fimmu.2022.845514

**Published:** 2022-02-11

**Authors:** Sohini S. Bhatia, Suresh D. Pillai

**Affiliations:** ^1^ National Center for Electron Beam Research, an International Atomic Energy Agency (IAEA) Collaborating Center for Electron Beam Technology, Texas A&M University, College Station, TX, United States; ^2^ Department of Food Science and Technology, Texas A&M University, College Station, TX, United States

**Keywords:** ionizing radiation, vaccines, electron beam, gamma irradiation, killed vaccines

## Abstract

Given the current pandemic the world is struggling with, there is an urgent need to continually improve vaccine technologies. Ionizing radiation technology has a long history in the development of vaccines, dating back to the mid-20^th^ century. Ionizing radiation technology is a highly versatile technology that has a variety of commercial applications around the world. This brief review summarizes the core technology, the overall effects of ionizing radiation on bacterial cells and reviews vaccine development efforts using ionizing technologies, namely gamma radiation, electron beam, and X-rays.

## Introduction

Vaccination is a cornerstone of public health measures. It promotes human and animal health as well as prevents the spread of communicable diseases in humans and animals. Over one hundred vaccines are currently licensed for human use in the United States (1). Despite this, many infectious diseases, such as Covid-19, HIV, Influenza, Malaria, and Tuberculosis continue to cause severe illness and death globally. In the feed and livestock animal industries, the use of antibiotic growth promoters has been substantially reduced due to fears of multi-drug resistant bacteria, (2–5). However, with the ban of antimicrobial usage, therapeutic usage of antimicrobials increased in Denmark by 33.6% (6) and mortality in weaning pigs increased by 1.5% (2). The resurgence of previously controlled infections and diseases have led to the intensive investigation and commercialization of multiple methods to control and improve animal health, with vaccinations being the most common (3, 7–9).

Current vaccine technologies have their advantages and disadvantages. Live vaccines often elicit strong immune responses, but a balance between attenuation, safety, and protection must be struck. Vaccination with attenuated strains has often been successful, although this option is not suitable for some diseases (10–13). A disadvantage of attenuated vaccines is the fear of regained virulence. Inactivated, or killed vaccines are inactivated using chemicals such as formalin, diethylpyrocarbonate and β-propiolactone. Although there are reduced safety risks associated with chemically inactivated vaccines, they often exhibit reduced immunogenicity due to damaged antigenic epitopes. Toxoids, recombinant vaccines, as well as subunit vaccines are typically considered safe because attenuation is induced by deletions preventing the strain from overgrowing and causing disease (14). The disadvantage of sub-unit vaccines is that only a singular antigen or at times multiple antigens are presented, generally limiting the cross-protective ability of such vaccines.

Given increased urbanization, climate change and close interaction of animals and humans, there is a continuous need to evaluate vaccine technologies to deal with epidemics, pandemics, and rapidly emerging infectious virus variants. The vaccine technologies should be robust and capable of dealing with multiple pathogens, their possible variants and host species (15). Ionizing radiation technology has benefitted society for over 65 years. Legacy nuclear technologies based on radioactive isotopes such as cobalt-60 and cesium-137 have resulted in significant benefits to human and animal health and agriculture. Besides radioactive isotope based ionizing radiation technology, electron beam (eBeam) and X-ray technologies have grown rapidly in the last decade and are now becoming widely used for a variety of commercial applications. The overall objective of this brief review is to summarize the history and the advances of using ionizing radiation technology for developing vaccines against infectious diseases.

## Principles of Ionizing Radiation

Ionizing radiation is defined as energy capable of removing electrons from atoms and, thereby, causing ionization. The three main ionizing radiation technologies are gamma radiation technology (based on photons), electron beam (eBeam) technology (based on electrons), and X-rays (based on photons) (16). Gamma rays are electromagnetic radiation composed of photons emitted from the nucleus of a radioactive isotope. In most commercial settings, the isotope source is cobalt-60. In some instances, gamma rays are produced from cesium-137 as well. Electron beam (eBeam) technology is based on highly energetic electrons that are produced from regular electricity using industrial equipment called “eBeam accelerators”. X-rays are also electromagnetic radiation composed of photons. However, they are generated using energetic electrons from accelerators which are allowed to strike an extremely dense metal such as tantalum or tungsten resulting in the formation of X-ray photons. Cobalt-60 is a radioactive isotope and, therefore, it is of serious security concerns. Also, due to increasing cobalt-60 costs, its stringent safe-guarding requirements, and ultimate disposal needs and costs, this legacy technology is quickly becoming commercially unsustainable. Commercially, gamma radiation technology is being quickly replaced with accelerator-based technologies, namely eBeam and X-ray technologies (16, 17). From a commercial perspective, eBeam technology is an attractive technology because of its relatively overall lower costs and relative ease of adoption. One of the key attractive features of eBeam and X-ray technologies is that they are switch- on/switch-off technologies meaning that they can be switched off when not in use. This is in direct contrast to radioactive isotopes such as cobalt-60 where the emission of gamma ray photons cannot be switched off.

Today, eBeam and X-ray technologies are commercial off the shelf technologies with a diverse array of energy and beam power configurations. In commercial settings, eBeam irradiation is generated using accelerators. In these accelerators, electrons generated from commercial electricity are accelerated to approximately 99.999% of the speed of light resulting in electron energies up to 10 MeV (Mega electron volts) (18). These highly energetic electrons are then focused and pulsed uniformly over a material, solid or liquid (16, 18). When the electrons interact with a molecule leading to its ionization, the ejected electron becomes energized, going on to interact with and ionize an adjacent molecule. This chain reaction continues until the energy has fully dissipated (18). High energy eBeam technology is also currently used in the food and medical device industry for its ability to either pasteurize products or achieve complete sterility. In the food industry, this technology is regularly used for phytosanitary treatment, shelf-life elongation, pathogen inactivation, and occasionally terminal sterilization (16, 17). In the medical device industry, this technology is used to sterilize single-use medical devices and laboratory consumables (19).

## Effect of Ionizing Radiation Exposure on Microorganisms

Ionizing radiation inactivates microorganisms through direct and indirect methods. Direct damage is caused as a result of interactions between energetic electrons or photons and the molecules within an organism, while indirect damage is caused as a result of interactions with products of water radiolysis (18, 20, 21). When an energized electron from an accelerator (or a gamma photon emitted from a radioactive isotope) interacts with a material, molecules are ionized, ejecting electrons from the outermost valence shells. These ejected electrons in turn cause a cascade of similar ionization events on adjoining atoms until all its energy is fully dissipated. In microorganisms, DNA is the largest molecule, therefore, resulting in it being the primary target of direct ionization events. The ionization of DNA results in the cleavage of the phosphodiester bonds along the DNA backbone. While single-stranded breaks are repairable, extensive double stranded breaks are much harder for an organism to repair and overcome. Due to excessive shearing of the nucleic acid, the microorganism is ultimately inactivated (21). The other major target of ionizing radiation in a microorganism is its cellular water content, leading to the production of radiolytic species. Radiolysis of water generates a diverse array of highly reactive, but short lived free radical species such as hydroxyl radicals, hydrogen peroxide, hydrogen, hydrated electrons, and hydrated protons. The summary equation for water radiolysis is presented below (Equation 1) with the quantity of each product per 100 eV of energy absorbed shown in parenthesis.


Equation 1 $$\begin{array}{l} {\text{e}}^{-}+{\text{H}}_{2}\text{O}\to^{*}\text{OH}(2.7)+{\text{e}}_{\text{aq}}^{-}(2.7){+}^{*}\text{H}(0.55)\\ +{\text{H}}_{2}(0.55)+{\text{H}}_{2}{\text{O}}_{2}(0.71)+{\text{H}}_{3}{\text{O}}^{+}(2.7) \end{array}$$

The damage to the cellular components often results indirectly from the interaction of these reactive species as opposed to the direct incident electrons. Hydroxyl radicals (*OH) are extremely short lived. However, during their short time, they can cause significant damage to molecules in their immediate surroundings (22). Superoxide radicals $\left({\text{O}}_{2^{-}}^{*}\right)$ are also generated by the radiolysis of water, and it is hypothesized that these molecules accumulate within a microbial cell causing severe damage to proteins such as enzymes with exposed iron-sulfur clusters (23, 24). Additionally, methionine and cysteine have been shown to be especially susceptible to ionizing radiation (25). Superoxide radicals also react with endogenous nitric oxide within a cell, forming reactive nitrogen species (RNS) such as a peroxynitrite anion (ONOO^-^), nitrogen dioxide $\left({\text{NO}}_{2}^{*}\right)$ , and dinitrogen trioxide (N_2_O_3_), which cause further damage to the DNA and are the primary agents of damage to proteins within bacterial cells (26). This protein damage can have significant effects on the microorganism’s ability to function. Taken together, direct and indirect mechanisms of damage lead to the inactivation of microbial cells due to the high number of single and double strand breaks (21). Assuming a hypothetical genome size of 3.5 million base pairs, a dose of 1 kGy would cause approximately 200 single stranded breaks and 14 double stranded breaks, per copy of a bacteria’s genome (18, 27). This extent of DNA damage is irreparable in most microorganisms, resulting in their inactivation due to the inability of the DNA to replicate, thereby, resulting in the microbial population being unable to reproduce. This damage done to microorganisms is extremely rapid. Direct damage due to chemical bonds cleavage is estimated to occur within 10^-14^ – 10^-12^ seconds of exposure. Within one picosecond (10^-12^ s), superoxide and hydrogen peroxide radicals are formed. By about 1 millisecond after exposure, the reactions of most reactive species are hypothesized to be complete (25, 28).

While microbial cells cease to multiply due to damage to their nucleic acids, multiple studies have demonstrated that their cellular membrane remains intact even after exposure to ionizing radiation. It needs to be pointed out the how microbial cells respond to ionizing radiation can be extremely varied depending on the microorganism in question and the ionizing radiation dose applied to the cells. Studies conducted in our laboratory demonstrate that eBeam exposure even at lethal doses does not compromise the bacterial cellular membrane as observed using microscopy (29–33). Similarly, gamma irradiation has also been shown to cause no damage to bacterial cell membranes at lethal doses (34–36). Furthermore, there is now significant evidence that in cells treated with lethal doses of ionizing radiation, there is residual metabolic activity after treatment (33, 35, 37–41). For example, in *Escherichia coli* K-12 metabolic activity of *E. coli* was sustained for up to nine days following treatment, as demonstrated using AlamarBlue™ as well as ATP assays (33). Other studies have demonstrated that gamma radiation also does not significantly hinder cellular functions. Gamma irradiated cells maintained oxidative function and the ability to continue nucleic acid and protein synthesis (35, 38). Furthermore, metabolic activity persists, despite several double stranded breaks of the cell’s genome. Researchers hypothesize that there are portions of genomes which are still intact, enough to sustain cellular functions (35, 39, 42). Bacterial cells exposed to eBeam exposure exhibit similar features. Studies examining the metabolomic state of inactivated *E. coli* and *Salmonella* Typhimurium have shown that immediately after treatment, cells are metabolically active with metabolomic fluxes continuing even 24 hours after eBeam treatment (43). Nevertheless, the ability of microbial cells to continue their metabolic activity even after physical damage to their nucleic acids is a scientific conundrum that is worthy of deeper investigation. Taken together, this state in microbial cells where the cells cannot multiply yet remain metabolically active can be termed as a Metabolically Active, yet Non-Culturable (MAyNC) state. In vaccinology, the term that is often used especially with irradiated malarial sporozoites is “Metabolically active, non-replicating”. This state has potential broad applications in vaccine development. MAyNC cells are inactivated, but maintain cell membrane integrity, and therefore, function as a killed vaccine. The biological significance of residual metabolic activity on the potency of the vaccine is yet to be completely understood. Because ionizing radiation maintains membrane integrity, MAyNC cells may be specifically well-suited for vaccines against pathogens that require immune recognition of multiple antigenic epitopes. Furthermore, due to the growing availability of eBeam and X-ray technologies which can be installed inline to the manufacturing process, the ability to generate MAyNC cells of varying potency can be extremely valuable for vaccine development.

## History of Vaccines Using Ionizing Radiation

The use of ionizing radiation as a method to attenuate or inactive microorganisms for the use as vaccines is not novel, with reports of gamma and x-ray-inactivated vaccine research dating back to the mid-20^th^ century (44–50). The advantage of ionizing-radiation vaccines, or radio-vaccines, is that because they are inactivated, they are able to retain immunogenicity even when stored at non-refrigerated conditions potentially eliminating the need for cold-chain to preserve vaccine potency (31, 51, 52). The ability to store vaccines at ambient or refrigerated storage (as compared to frozen storage) can translate to significantly lower overall costs for vaccine transportation and distribution. The ability to distribute vaccines without the need for cold chain distribution also increases vaccine access in remote areas (53, 54). Importantly, eBeam and X-ray technologies are scalable, with the capability to inactivate large quantities of preparations (55).

Due to the vast commercial capabilities, numerous patents related to “radio-vaccines” have already been filed (**Table 1**). Interest in radio- vaccines has increased significantly recently, with investigations into the creation of vaccines for bacterial, viral, and protozoan diseases (**Table 2**). While many of the researched vaccine candidates are based on gamma-irradiation, there is significantly less research conducted on eBeam or X-ray inactivated vaccines. This limited amount of information could be attributed to the relatively recent commercial availability of eBeam and X-ray technologies. Among all the research conducted on radio-vaccines, the most progress has been on *Plasmodium* sporozoites attenuated with irradiation to protect against malaria. First examined in 1967 using x-ray irradiation, this idea has evolved considerably over the last 50+ years to its current iteration in phase 2 clinical trials using gamma-attenuated sporozoites (75, 76, 93–97). Studies using gamma-irradiated *Listeria monocytogenes* have demonstrated that unlike other inactivation methods such as heat or formalin, irradiation better maintained antigenic properties and stimulated robust T cell responses (59).

**Table 1:** A selection of patents relating to radio-vaccines.

Patent #	Country	Year	Status^a^	Title
US3657415A	USA	1969	Expired	Canine hookworm vaccines
DE3853854T2	Germany	1988	Expired	Vaccine against group b *Neisseria meningitidis*, gammaglobulin and transfer factor
AU706213B2	Australia	1996	Ceased	Method for obtaining a vaccine with wide protective range against group b *Neisseria meningitidis*, the resulting vaccine, gammaglobulin and transfer factor
AU6320001A	Australia	2001	Published	Gamma irradiation of protein-based pharmaceutical products
KR20030034517A	South Korea	2001	Granted	*Burkholderia gladioli* k4 having antifungal activity, preparation method of its mutant by gamma radiation and the mutant thereof
US20060147460A1	USA	2002	Granted	Anticancer vaccine and diagnostic methods and reagents
KR101173871B1	South Korea	2004	Granted	Modified free-living microbes vaccine compositions and methods of use thereof
US20050175630A1	USA	2004	Abandoned	Immunogenic compositions and methods of use thereof
US8173139B1	USA	2009	Granted	High energy electron beam irradiation for the production of immunomodulators in poultry
CA2733356C	Canada	2009	Granted	Influenza vaccines
US8282942B2	USA	2010	Granted	*Toxoplasma gondii* vaccines and uses thereof
US20130122045A1	USA	2010	Abandoned	Cross-protective influenza vaccine
US20150209424A1	USA	2011	Abandoned	Inactivated varicella zoster virus vaccines, methods of production, and uses thereof
JP2014520117A	Japan	2012	Granted	Vaccine composition comprising inactivated chikungunya virus strain
AU2012211043B2	Australia	2012	Published	Combination vaccines
US10080795B2	USA	2013	Granted	Method for inactivating viruses using electron beams
WO2014155297A2	WIPOb^b^	2014	Published	Systems and methods for viral inactivation of vaccine compositions by treatment with carbohydrates and radiation
WO2014165916A1	WIPO^b^	2014	Published	Methods and compositions for inducing an immune response
DE102015224206B3	Germany	2015	Granted	Irradiation of biological media in transported foil bags
KR20180036987A	South Korea	2016	Published	Vaccine composition
DE102016216573A1	Germany	2016	Published	Inactivation of pathogens in biological media
WO2018167149A1	WIPO^b^	2018	Ceased	Method for irradiating mammalian cells with electron beams and/or x-rays
WO2019191586A2	Canada	2019	Published	Irradiation-inactivated poliovirus, compositions including the same, and methods of preparation
WO2020069942A1	WIPO^b^	2020	Published	Method for inactivating biologically active components in a liquid

a Status as of November, 2020; World Intellectual Property Organization.

**Table 2:** List of radio-vaccines against bacterial, viral, and protozoan pathogens.

Type of Pathogen	Pathogen	Inactivation Method	Inactivation Dose	Model	Notes	Source
Bacteria	*Brucella abortus*	Gamma	4 kGy	Mice	Irradiated strains induced less of an immune response than live strains	(56)
Bacteria	*Brucella abortus*	Gamma	3 kGy	Mouse	Antigen specific Th1 response	(34)
Bacteria	*Brucella abortus*	Gamma	2.5 kGy	Mice	Stimulated IFN-gamma and Th1 cells	(57)
Bacteria	*Brucella abortus*	Gamma	3.5 kGy	Mice	Protective upon challenge	(58)
Bacteria	*Brucella abortus, B. melitensis, and B. suis*	Gamma	3.5 kGy	Mice	Protective upon challenge	(36)
Bacteria	*Brucella melitensis*	Gamma	3.5 kGy	Mouse	Cytotoxic T cell response and protective against challenge	(35)
Bacteria	*Listeria monocytogenes*	Gamma	6 kGy	Mouse	Induced protective T cell responses	(59)
Bacteria	*Mannheimia haemolytica*	Gamma	2-20 kGy	Rabbit	Protection upon challenge	(60)
Bacteria	*Orientia tsutsugamushi*	Gamma	2 kGy	Mice	Partially protective upon challenge	(61)
Bacteria	*Orientia tsutsugamushi*	Gamma	3 kGy	Mice	Protective upon challenge	(62)
Bacteria	*Pasteurella tularensis*	X-ray	10 kGy	Mice	Partially protective upon challenge	(63)
Bacteria	*Rhodococcus equi*	Electron Beam (High Energy)	4-5 kGy	Horse	Produced cell-mediated and upper respiratory mucosal immune response	(30)
Bacteria	*Rhodococcus equi*	Electron Beam	5 kGy	Horse	Not protective upon challenge	(64)
Bacteria	*Rodentibacter pneumotropicus*	Electron Beam (Low Energy)	20 kGy	Mice	Protective upon challenge and reduced colonization	(65)
Bacteria	*Salmonella* Enteriditis	Electron Beam (High Energy)	2.5 kGy	Chicken	Protective upon challenge and reduced colonization	(66)
Bacteria	*Salmonella* Typhimurium	Electron Beam (High Energy)	2.5 kGy	Chicken	Heterophil-mediated innate immune response	(67)
Bacteria	*Salmonella* Typhimurium	Electron Beam (High Energy)	7 kGy	Mouse	Stimulated innate immune markers and reduced colonization	(31)
Bacteria	*Salmonella* Typhimurium	Gamma	10-80 kGy	Chicken	Protective upon challenge	(68)
Bacteria	*Shigella dysenteriae*	X-ray	Not reported	Rabbits	Bacteria that were treated for a longer time were non-toxic and protective upon challenge	(44)
Bacteria	*Staphylococcus aureus*	Gamma	2.5-2.9 kGy	Mice	Induced specific antibody production, but not protective upon challenge	(69)
Bacteria	*Staphylococcus aureus*	Gamma	25-40 kGy	Mice	Induced B and T cell-dependent protection against challenge	(70)
Bacteria	*Streptococcus pneumoniae*	Gamma	12 kGy	Mice	Protection upon challenge mediated by B-cells and innate IL-17 response	(71)
Bacteria	*Streptococcus pneumoniae*	Electron Beam	25 kGy	Rabbit and Mice	Immunogenic and protective upon challenge	(72)
Protozoa	*Eimeria tenella*	Electron Beam (Low Energy)	0.1-0.5 kGy	Chicken	Partially protective upon challenge	(73)
Protozoa	*Eimeria tenella*	X-ray	0.2 kGy	Chicken	Protective upon challenge	(74)
Protozoa	*Plasmodium berghei*	X-ray	0.02-0.15 kGy	Mouse	Protective upon challenge	(75)
Protozoa	*Plasmodium falciparum*	Gamma	0.12-0.15 kGy	Human	Long-lasting protective immunity	(76)
Protozoa	*Plasmodium gallinaceum*	X-ray	0.005-0.2 kGy	Mosquito	Sporozoites from irradiated oocysts were non-infective	(49)
Virus	Human Respiratory syncytial virus (RSV)	Electron Beam (Low Energy)	20 kGy	Mice	Reduction in viral load upon challenge	(77)
Virus	Influenza A virus	Gamma	12.6 kGy	Mice	Induced cytotoxic T cells and protective upon against challenge	(78)
Virus	Influenza A virus	Gamma	10-40 kGy	Mice	Cross-reactive and cross-protective cytotoxic T cell responses	(79)
Virus	Influenza A virus	Gamma	10 kGy	Mice	Protective upon challenge; freeze-drying did not affect cross-protective immunity	(80)
Virus	Influenza A virus	Gamma	50 kGy	Mice	Intranasal vaccination conferred complete protection	(81)
Virus	Influenza A virus	Electron Beam (Low Energy)	30 kGy	Mouse	Elicited a protective immune response	(82)
Virus	Influenza A virus	Electron Beam	25-40 kGy	Nonhuman primate	Elicited seroconversion	(51)
Virus	Influenza A virus	Gamma	10 kGy	Mice	Protective upon heterotypic challenge	(83)
Virus	Middle Eastern Respiratory Virus (MERS)	Gamma	50 kGy	Mice	Caused lung immunopathology upon challenge	(84)
Virus	Polio Virus	Gamma	45 kGy	Mice	Protective upon challenge	(85)
Virus	Rotavirus	Gamma	50 kGy	Mice	Induced a specific neutralizing-antibody response	(86)
Virus	Severe Acute Respiratory Syndrome coronavirus 2 (SARS-CoV-2)	Gamma	50 kGy	Mice	Adjuvanted vaccine elicited T and B cell responses	(52)
Virus	SARS-CoV-2	Gamma	25 kGy	Mice	Humoral and cellular immune response, induced neutralizing antibodies	(87)
Virus	Vaccinia virus	Gamma	0-15 kGy	Rabbit	Inactivated virus was immunogenic	(48)
Virus	Venezuelan Equine Encephalitis Vaccine	Gamma	80-100 kGy	Guinea Pig	Protective upon challenge	(88)
Virus	Venezuelan Equine Encephalitis Vaccine	Gamma	50 kGy	Mice	Protective against subcutaneous challenge and partially protective against aerosol challenge	(89)
Virus	White Spot Syndrome Virus	Electron Beam	13 kGy	Shrimp	Protective upon challenge	(90)
Virus	Zaire ebola virus	Gamma	100 kGy	Nonhuman primate	Not protective upon challenge	(91)
Virus	Zaire ebola virus	Gamma	60 kGy	Nonhuman primate	Not protective upon challenge	(92)

## Immune Responses to Radio-Vaccines

In multiple studies investigating the immune response to gamma-irradiated *Brucella* spp., investigators found that gamma-irradiated cells were metabolically active and inactivated cells were able to induce a significant cellular immune response and were protective when challenged (34–37, 56, 98). Furthermore, gamma-irradiated cells have even exhibited an ability to act as an adjuvant, increasing the immune response to co-administered antigens (71). A significant amount of research has been conducted on the development of a gamma-inactivated influenza vaccine, demonstrating that this vaccine is effective in eliciting a strong antigen-specific antibody response as well as protecting mice from challenge with heterologous influenza virus (27, 80, 83).

Electron beam (eBeam) technology has been investigated as a method to generate vaccine-like immunomodulators against *Salmonella* Typhimurium using a mice model (31). This concept has been expanded to demonstrating the immunomodulatory and protective effects of eBeam-inactivated *Salmonella* Enteriditis and Typhimurium in chickens and *Rhodococcus equi* in neonatal fouls (29–32, 40, 41, 64, 67). This concept is now been expanded to include the use of low energy eBeam as an inactivation technology for vaccine development with considerable success (73, 77, 82).

## Role of Adjuvants

For a vaccine formulation to be effective upon challenge, it must be able to induce a prolonged and protective immune response. Live attenuated vaccines that retain their ability to replicate with a host, naturally eliciting a strong CD8+ and CD4+ T cell response, as well as a strong humoral response, while inactivated vaccines often require the assistance of an adjuvant to help the vaccine elicit a stronger immune response in the host. An adjuvant is technically defined as a component that is added to vaccine to enhance an immune response, and typically provides the benefits of increased antibody titers and an increased speed, breadth, and duration of an immune response. Because radio-vaccines are unable to replicate within a host, it has been proposed that their immunogenic potential has to be enhanced by the addition of an adjuvant. There are several reports about coupling radio-vaccines with experimental and commercially available adjuvants. Bayer et al. tested four different adjuvants in combination with Respiratory syncytial virus inactivated with low energy electron beam: Alhydrogel (alum based), MF59 (squalene based), QuilA (saponin based), and Poly IC : LC (synthetic double-stranded RNA based) (77). In their study, strong immune responses and significant reductions in viral loads were detected after immunization and subsequent challenge, although the poly IC : LC adjuvanted vaccine elicited lower titers of neutralizing antibodies than the other adjuvanted vaccines tested (77). Substantial humoral and cellular responses were observed when a gamma-inactivated polio vaccine candidate was combined with an alum adjuvant and when a gamma-irradiated HIN1 vaccine was co-administered with a plasmid encoding mouse interleukin-28B (99, 100). Gamma-inactivated SARS-CoV-2 also benefited from the addition of a GM-CSF adjuvant in order to induce a T cell response (52).

## Conclusions

Though ionizing radiation has been researched as a vaccine technology for nearly a century, only recently have vaccines utilizing ionizing radiation reached commercial development. The general lack of interest in radio-vaccines could be attributed to advances in cloning technologies, mRNA vaccines and gene editing technologies. The recent availability of small footprint, low energy eBeam and X-ray equipment could, however, spur the development of radio-vaccines once again. Commercialization of eBeam and X-ray technologies for the medical device, food, and other industrial applications has led to a decrease in overall technology costs and an increase in technology availability (101). This review highlights the potential of ionizing radiation as a vaccine technology suitable against several pathogens causing diseases in various hosts species. This has been most recently demonstrated in the rapid development of vaccine candidates in response to the COVID-19 pandemic, caused by the virus SARS-CoV-2. Radio-vaccines have even been investigated as a response to previous outbreaks of SARS and MERS, and it was hypothesized that ionizing radiation could be used to rapidly produce a vaccine for SARS-CoV-2 (84, 102–104). Gamma-inactivated SARS-CoV-2 combined with GM-CSF as an adjuvant has demonstrated ability to induce neutralizing antibodies as well as a strong T and B cell response (87, 105).

## Author Contributions

Major portions of this manuscript have been previously included in a doctoral dissertation by SB (106). SP was involved in writing and editing the manuscript. All authors contributed to the article and approved the submitted version.

## Funding

This work was supported funds from the USDA-NIFA program administered by Texas A&M AgriLife Research H-87080 as well as funds through contracts from the Pacific Northwest National Laboratories (PNNL).

## Conflict of Interest

The authors declare that the research was conducted in the absence of any commercial or financial relationships that could be constructed as a potential conflict of interest.

## Publisher’s Note

All claims expressed in this article are solely those of the authors and do not necessarily represent those of their affiliated organizations, or those of the publisher, the editors and the reviewers. Any product that may be evaluated in this article, or claim that may be made by its manufacturer, is not guaranteed or endorsed by the publisher.
